# Maternal and newborn care during the COVID-19 pandemic in Kenya: re-contextualising the community midwifery model

**DOI:** 10.1186/s12960-020-00518-3

**Published:** 2020-10-07

**Authors:** Rachel Wangari Kimani, Rose Maina, Constance Shumba, Sheila Shaibu

**Affiliations:** 1 School of Nursing and Midwifery, Aga Khan University, PO Box 39340, Nairobi, 00623 Kenya; 2 Department of Population Health, Aga Khan University, PO Box 39340, Nairobi, 00623 Kenya

**Keywords:** COVID-19, Community health, Midwifery, Kenya, LMIC, Africa, Pandemic, Maternal, Neonatal, Community Interventions, Coronavirus, Pregnant women

## Abstract

Peripartum deaths remain significantly high in low- and middle-income countries, including Kenya. The COVID-19 pandemic has disrupted essential services, which could lead to an increase in maternal and neonatal mortality and morbidity. Furthermore, the lockdowns, curfews, and increased risk for contracting COVID-19 may affect how women access health facilities. SARS-CoV-2 is a novel coronavirus that requires a community-centred response, not just hospital-based interventions. In this prolonged health crisis, pregnant women deserve a safe and humanised birth that prioritises the physical and emotional safety of the mother and the baby. There is an urgent need for innovative strategies to prevent the deterioration of maternal and child outcomes in an already strained health system. We propose strengthening community-based midwifery to avoid unnecessary movements, decrease the burden on hospitals, and minimise the risk of COVID-19 infection among women and their newborns.

## Background

The COVID-19 pandemic caused significant disruption of essential health services in sub-Saharan Africa. As COVID-19 continues to spread in Africa, health resources have been diverted to focus on general population needs rather than the specific needs of vulnerable groups, such as pregnant women and their children. These modifications in resource allocation may cause an increase in maternal and newborn deaths [1]. Given the high burden of maternal and neonatal mortality in sub-Saharan Africa, there is an urgent need for innovative strategies to prevent the deterioration of maternal and child outcomes in already strained health systems.

Limited information is available about the pathophysiological effect of SARS-CoV-2 on pregnant women. However, emerging evidence indicates that pregnant women are not at a higher risk for severe COVID-19 illness than other population groups [2, 3]. To date, most reports of COVID-19 cases in pregnancy were either asymptomatic or self-limiting pneumonia [4]. Nevertheless, immunological and physiological adaptations during pregnancy could make women more vulnerable to SARS-CoV-2 infection than the general populace [5]. Furthermore, there have been reports of severe illness among pregnant women with comorbidities, such as diabetes [6]. In addition, previous human coronaviruses (e.g. pandemic influenza and SARS) were associated with increased maternal and neonatal deaths [7, 8].

Although pregnancy is ordinarily considered a normal physiological and developmental milestone, the increased stress women can experience during public emergencies may result in preterm birth, low birth weight, and other high-risk conditions. Recent reports from China [3, 9] and the United States of America [10] showed that a majority of pregnant women that were positive for COVID-19 delivered via caesarean section because of foetal distress [11]. There is limited evidence concerning the possible maternal-foetal transmission and neonatal infection, and case reports have described COVID-19 in healthy newborns as a mild self-limiting disease [3, 4, 12].

## Impact of COVID-19 on maternal and newborn care

Scientists and public health officials are accelerating efforts to prevent, treat, and control COVID-19. However, minimising exposure to SARS-CoV-2 remains the only stratagem to reduce the risk of infection. Social isolation presents additional challenges, as current maternal and child health (MCH) guidelines advocate for women to attend regular antenatal (ANC) visits and deliver in health facilities. Despite the implementation of social distancing measures in hospitals, emerging evidence indicates that contagion is worse when healthcare is centralised [13–16]. Therefore, it is important to explore alternative measures that target the community rather than just healthcare facilities.

In Kenya, there have been reports of decreased antenatal attendance, immunisations, and hospital deliveries, along with an increase in stillbirths during COVID-19 [17–19]. This decline may be attributable to restricted access to health facilities arising from city lockdowns and curfews imposed by the government, where pregnant women and their companions fear harassment and arrest by the police. In addition, fear of contracting COVID-19 may keep many women from attending reproductive health services. Women reported similar sentiments concerning fear of infection risk at health facilities during the recent Ebola pandemic [1, 20].

## Policy and service responses to COVID-19

Government directives and hospital policies limit the number of family members accompanying expecting women to hospitals to prevent contagion. There is also a separation of COVID-19-positive women from their newborns, instead of room-in as usual for these mothers and newborns [21]. Though the separation may minimise the risk of SARs-CoV-2 transmission from mother to infant during the hospital stay, this precaution may do more harm than good [22, 23]. Evidence shows that separation disrupts skin-to-skin care and breastfeeding and is associated with added physiologic stress to both the mother and infant [24, 25]. Moreover, neonates born to COVID-19-positive mothers have to be isolated from other babies to stop the spread of COVID-19 in newborn nurseries. This requires extra healthcare providers to care for isolated mothers and newborns, which places increased pressure on an already burdened health system. Though the Kenyan government has pledged to hire new healthcare workers, the goal of this new workforce is to fill human resource gaps in the COVID-19 pandemic, and it remains unclear how this would impact MCH services.

In these extraordinary times, there is benefit from avoiding sources of infection and limiting health facility use to those who need it; therefore, should we not re-evaluate whether it is appropriate for all women to give birth in health facilities? Further, low- and middle-income countries (LMICs) are encouraged to initiate public health innovations that are culturally acceptable [26]. Research shows that risk perception influences where women give birth [27]. Given the complexities posed by COVID-19, it is prudent to consider alternatives to existing delivery options for women.

This article proposes a strategic response to maternal and newborn care in Kenya in the context of COVID-19. We recommend expanding and strengthening the existing community midwifery model (CMM), integrating community health workers (CHWs) and informal community networks, and creating midwifery centres run by qualified midwives that are closer to communities. We consider these strategies to offer a viable long-term plan that will reduce the burden on hospitals and decrease COVID-19 infection rates among pregnant women and their newborns.

## CMM in Kenya

As in other LMICs, maternal mortality in Kenya has shown a minimal decline over the years. WHO trends (1990–2015) placed Kenya among the countries with high maternal mortality and neonatal mortality rates, at 342/100 000 and 19/1000 live births, respectively [28]. It has been challenging to improve maternal and newborn outcomes because of service delivery gaps, cultural beliefs, lack of social support networks, and inaccessible emergency maternal obstetric care. Furthermore, according to the Confidential Enquiry Maternal Deaths 2017 report, 20% of maternal deaths had an avoidable community determinant [29]. This report emphasised the importance of community-based interventions to improve maternal and neonatal outcomes.

Despite the frequency of public health emergencies in Kenya (e.g. floods, terrorism, and ethnic violence), there are no adequate disaster plans that address how pregnant women will deliver during emergencies. In this prolonged COVID-19 crisis, women will continue to become pregnant and give birth. Furthermore, there are reports of high rates of teenage births during the pandemic lockdown [30], which is likely to increase the workload for maternal and newborn healthcare. Spikes in adolescent pregnancy were also reported during the Ebola and Zika virus crisis leading to adverse maternal outcomes [1]. Despite the ongoing COVID-19 crisis, pregnant women deserve a safe and humanised birth with a priority on physical and emotional safety for the mother and the baby. An effective strategy depends on stakeholders innovating evidence-based strategic plans to ensure safe care during childbirth.

Under normal conditions, around 61% of pregnant women in Kenya give birth with the assistance of a skilled attendant [28]. During the COVID-19 crisis, barriers such as lockdowns, curfews, and risk for infection prevented women from accessing health facilities. The resulting increase in home deliveries offers an opportunity to introduce critical birthing, postnatal, breastfeeding, immunisation, and family planning services into women’s homes. A community midwifery approach can close the utilisation gap and optimise MCH services during this crisis.

The CMM is an innovative community-based health intervention that involves deploying skilled midwives residing in their communities to take critical maternal health services into women’s homes [31]. The CMM was introduced as part of the National Health Sector Strategic Plan II, which emphasised the promotion of community health [32]. This strategic plan was revised in 2012, and a community health strategy was incorporated into the Kenya Health Policy (2012–2030) and Vision 2030. These documents highlighted the significant role of community midwives in providing culturally acceptable, skilled birthing services. Although the momentum for community midwifery in Kenya seems to have slowed given the paucity of the current literature, COVID-19 is an opportunity to revisit the critical role community midwives can play in bridging the gap in skilled deliveries.

As Kenya attempts to revitalise its primary healthcare delivery system to provide universal healthcare, leveraging past success is a good starting point. Evidence from studies conducted in Kenya shows that community midwives improved ANC attendance, family planning, HIV services, skilled birth attendance, postnatal care, and exclusive breastfeeding [33–36]. However, the continued success of community midwifery depends on their recognition as public health workers and adequate remuneration for services rendered by government insurance schemes.

In Kenya, nurses and midwives provide the majority of peripartum care. COVID-19 has strained human resources for health. This shortage presents an option to consider integrating CHWs and volunteers into the CMM framework. Research in rural areas demonstrated that strengthening collaboration with CHWs and informal community networks improved ANC and normalised vaginal births [31, 33, 36, 37]. Evidence from LMIC shows that community-based interventions are effective in enhancing MCH services [38]. However, a significant risk of home-based care is the delay in accessing skilled care resulting in complications, due to the lack of knowledge on danger signs [39, 40]. The delay may also occur due to the limited number of midwives available in the community [31]. A new policy would concentrate on standardising high-quality community-based MCH interventions through optimal timing of home visits and referrals [38, 41]. It is essential to continue to address social and gender norms that pose barriers to accessing skilled childbirth services even when these are available at the community level [42]. Further, the country needs to invest in improving the number, training, and supervision of midwives to improve safety and reduce harm [42].

In low-resource areas where technology is limited, monitoring maternal and foetal conditions is challenging; however, midwives can screen pregnant women in their localities for complications and initiate referrals for specialised care for high-risk women. Readily available transport mechanisms need to be identified within communities for referrals during crises, especially in rural areas. Additionally, there needs to be a mechanism for midwives to manage complications if birth is imminent or it is not possible to transfer a pregnant woman to a facility when complications are anticipated. Childbirth kits should also be available in the event of a delay in transporting a pregnant woman [33, 37]. In addition, all community midwives and CHWs should be equipped with the necessary personal protective equipment to minimise their exposure to SARS-Cov-2 [13].

## Recommendations

As reported in pandemics in West Africa [1, 43], crises exacerbate maternal and neonatal mortality. Lessons learned from previous crises in Kenya that impacted the healthcare system highlighted the need to prioritise maternal and newborn care [35, 44]. Therefore, bold, long-term, and culturally acceptable measures are needed to address maternal and newborn care during this prolonged COVID-19 crisis. In the current pandemic, there is benefit from avoiding sources of infection and decongesting hospitals by offering midwifery services in the community. Although some pregnant women will still require hospitalisation, healthy women should be kept in the community. There are direct effects on access to MCH services as a result of the added burden of caring for COVID-19 patients within healthcare settings and the diversion of resources away from MCH towards caring for COVID-19 patients elsewhere. Further, there are knock-on effects arising from the impact of precautionary policies regarding access to health facilities and services intended to enable safer healthcare delivery during the pandemic, as well as the impact of more sweeping social measures during the pandemic such as lockdowns and curfew. These effects highlight the need to innovate and shift from an exclusive focus on hospital-based maternal care to community-based interventions [14]. It is time to enhance the CMM in Kenya to create demand and improve access to and use of skilled birth attendance and postnatal services for vulnerable women in informal urban settlements and rural areas.

A CMM during the COVID-19 pandemic should include the following:

1. Dedicated community-based midwives working within their communities with proper infection prevention and control training to minimise the risk of infection to themselves and the community. We suggest community midwives be separated from health facilities caring for patients with COVID-19. Community midwives also need to be recognised and supported to be key providers of care at the community level through fair remuneration, supportive supervision, and appropriate resources.
2. Collaboration with existing informal social networks and incorporation of existing CHWs, community health extension workers, and civil societies into the community framework. It is also prudent to retrain and build community midwives and CHWs to strengthen community health linkages, prevent transmission of disease, and overcome context-specific barriers that prevent women from accessing quality maternal care.
3. Creating referral pathways and transport systems to strengthen facility-community linkages. A dedicated telephone line with direct access to community midwives should be established in each county to serve women and their families. Midwives can triage, offer advice, and refer women to maternal services as well as mental health, gender-based violence, and social services, as needed.
4. Development of long-term disaster plans that give clear guidelines that keep women and their newborns healthy in their communities without exposing them to unnecessary risks.

COVID-19 is a highly contagious disease with significant indirect ramifications for maternal and child health outcomes. Lockdowns and social distancing are needed to reduce transmission. However, given the economic impact of mitigation measures on the economy, the Kenyan government will soon have to lift these restrictions. This public health emergency requires solutions that move from facility-based care to outreach to the community. Although some pregnant women will require hospital admission, it is also possible for healthy women to safely give birth in communities with the help of skilled midwives. In turn, this would help to address the universal healthcare agenda. The COVID-19 pandemic is a prolonged crisis that needs a long-term plan to ensure women and newborns continue to receive culturally appropriate care.

## Data Availability

Data sharing is not applicable to this article as no datasets were generated or analysed during the current study.

## Abbreviations

ANC: Antenatal
MCH: Maternal and child health
LMIC: Low- and middle-income countries
CHWs: Community health workers
CMM: Community midwifery model
